# Activation of PyMT in β Cells Induces Irreversible Hyperplasia, but Oncogene-Dependent Acinar Cell Carcinomas When Activated in Pancreatic Progenitors

**DOI:** 10.1371/journal.pone.0006932

**Published:** 2009-09-07

**Authors:** Yi-Chieh Nancy Du, David S. Klimstra, Harold Varmus

**Affiliations:** 1 Program in Cancer Biology and Genetics, Memorial Sloan-Kettering Cancer Center, New York, New York, United States of America; 2 Department of Pathology, Memorial Sloan-Kettering Cancer Center, New York, New York, United States of America; Karolinska Institutet, Sweden

## Abstract

It is unclear whether the cellular origin of various forms of pancreatic cancer involves transformation or transdifferentiation of different target cells or whether tumors arise from common precursors, with tumor types determined by the specific genetic alterations. Previous studies suggested that pancreatic ductal carcinomas might be induced by polyoma middle T antigen (*PyMT*) expressed in non-ductal cells. To ask whether PyMT transforms and transdifferentiates endocrine cells toward exocrine tumor phenotypes, we generated transgenic mice that carry tetracycline-inducible *PyMT* and a linked *luciferase* reporter. Induction of *PyMT* in β cells causes β-cell hyperplastic lesions that do not progress to malignant neoplasms. When *PyMT* is de-induced, β cell proliferation and growth cease; however, regression does not occur, suggesting that continued production of PyMT is not required to maintain the viable expanded β cell population. In contrast, induction of *PyMT* in early pancreatic progenitor cells under the control of *Pdx1* produces acinar cell carcinomas and β-cell hyperplasia. The survival of acinar tumor cells is dependent on continued expression of *PyMT*. Our findings indicate that PyMT can induce exocrine tumors from pancreatic progenitor cells, but cells in the β cell lineage are not transdifferentiated toward exocrine cell types by PyMT; instead, they undergo oncogene-dependent hyperplastic growth, but do not require PyMT for survival.

## Introduction

The pancreas is a key regulator of glucose homeostasis and of protein and carbohydrate digestion [1]. It is composed of two major compartments, the endocrine pancreas and the exocrine pancreas. The endocrine pancreas, which regulates metabolism and glucose homeostasis, consists of five hormone-expressing cell types – α, β, δ, ε, and pp cells – that produce glucagon, insulin, somatostatin, ghrelin, and pancreatic polypeptide, respectively. Endocrine cells are arranged in clusters, called the islets of Langerhans, and most of the cells in each islet are β cells [2]. The exocrine pancreas is composed of the digestive enzyme-producing acinar cells and a branching network of ducts that direct the enzymes to the intestines.

A spectrum of pancreatic malignancies possess histological and molecular features that recapitulate to some degree the properties of their normal cellular counterparts, and rare pancreatic tumors with mixed differentiation have also been described [1], [3]. The cellular origins of pancreatic cancers have been debated. It is not clear whether different pancreatic tumor types arise from transformation of different target cells, from transformation followed by transdifferentiation, or from transformation of a common precursor, with the phenotypes determined by the differentiation effects of specific genetic alterations. Pancreatic cells have been shown to be susceptible to transdifferentiation: by ectopic expression of transcription factors *in vivo*, adult β cells can be transdifferentiated into α and pp cells [4], and exocrine cells can be transdifferentiated into β cells [5].

An early study indicated that murine pancreatic ductal adenocarcinomas can be generated by *in vitro* transduction of polyoma middle T antigen (*PyMT*) into the islets prepared from juvenile mice, but not from aged mice [6]. A subsequent report from our laboratory showed that delivery of an avian leukosis-sarcoma virus (ALSV)-based vector encoding *PyMT* or *cMyc* to newborn *elastase-tva*; *Ink4a/Arf*-null transgenic mice, in which the receptor for subgroup A avian leukosis virus, TVA, is expressed in multiple pancreatic cell types, induces different pancreatic lesions [7]. In those experiments, PyMT induced cystic papillary neoplasms with ductal differentiation and pancreatic intraepithelial neoplasia (PanIN) lesions, as well as carcinomas with mixed acinar and endocrine components, whereas cMyc induced endocrine neoplasms that express insulin. We could not determine, however, whether PyMT transforms multipotential progenitor cells, possibly including islet cell precursors, and guides them toward exocrine cell fates, or whether PyMT transforms only the cells that are already committed to exocrine cell fates.

To distinguish between these possibilities, we generated transgenic mouse lines with a tetracycline-inducible *PyMT* oncogene that could be turned on and off in different cell types and different stages of development by expressing a second transgene encoding a tetracycline-dependent regulatory protein under different cell-specific promoters. A *luciferase* reporter was co-activated in the *PyMT* transgene, so that transgene expression could be detected by *in vivo* bioluminescent imaging. Although *PyMT* is not implicated in human cancer, it remains an important experimental tool because its product is a potent oncoprotein, and stimulates at least two signaling pathways that are important in human cancers – the mitogen-activated protein kinase (MAPK) and phosphatidylinositol 3-kinase (PI3K) cascades [8].

Our studies suggest that the ability of *PyMT* to induce tumorigenesis is dependent upon the types of the pancreatic cells in which it is expressed. Conditional activation of *PyMT* in β cells led to irreversible non-malignant expansion of the β cell population, regardless of the developmental stage at which it was expressed. However, activation of *PyMT* in the common precursors of both exocrine and endocrine pancreatic cells induced lethal acinar cell carcinomas in some mice, as well as β-cell hyperplasia. Furthermore, although continued expression of *PyMT* is required for the survival of the acinar cell carcinoma cells, as is true for many types of oncogene-induced neoplasms in mice [9], [10], it is not required to sustain the survival of the hyperplastic β cells.

## Results

### Generation of mice with a Tet-regulated *PyMT* oncogene

To generate mice in which we could regulate the expression of *PyMT* in the pancreas, we used the tetracycline regulatory system. A *tet-o-PyMT-IRES-Luc* responder transgene was constructed carrying the *PyMT* gene downstream of *tet* operator sequences (details in Materials and Methods). To facilitate detection of transgene expression, this 5.8 kb piece of DNA encodes a bicistronic mRNA consisting of a *luciferase* reporter gene translated from an internal ribosome entry site (IRES) downstream of the *PyMT* coding region. Seven *tet-o-PyMT-IRES-Luc* transgenic founders (#1, 2, 20, 21, 23, 29, and 39) were identified by PCR genotyping among the 39 pups obtained from the microinjection of this transgene into C57BL/6 mouse eggs, and the transgene was transmitted to the progeny of all 7 founder lines. However, founder line #20 expressed luciferase ubiquitously without a tetracycline regulatory protein, as determined by *in vivo* bioluminescent imaging (data not shown). This line was not evaluated further.

### Activation of *PyMT* induces hyperplasia in the β cells of *RIP-rtTA*; *tet-o-PyMT-IRES-Luc* bitransgenic mice

**Figure 1:**
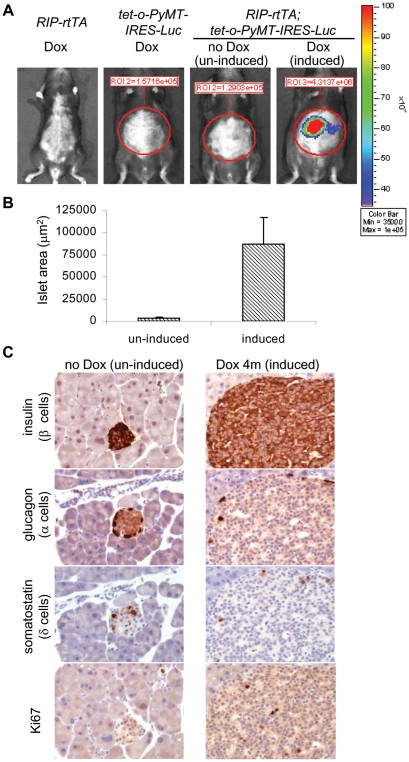
Expression of the *PyMT-IRES-Luc* transgene in the pancreata of *RIP7-rtTA; tet-o-PyMT-IRES-Luc* bitransgenic mice resulted in β-cell hyperplasia. (A) Transgene expression in *RIP7-rtTA; tet-o-PyMT-IRES-Luc* bitransgenic mice monitored by *in vivo* bioluminescent imaging. Control *RIP7*-rtTA and *tet-o-PyMT-IRES-Luc* mono-transgenic mice, and *RIP7-rtTA; tet-o-PyMT-IRES-Luc* bitransgenic mice that were and were not on a doxycycline diet for 4 months were subjected to bioluminescence. Significant luciferase activity was detected in the area over the pancreas only in the bitransgenic mouse on doxycycline (induced). The images are representative of more than 10 mice from each group. (B) Total area of islets in *RIP7-rtTA; tet-o-PyMT-IRES-Luc* bitransgenic mice that were not on doxycycline (un-induced) compared with islet area in animals that received doxycycline at 4 weeks of age for 4 months (induced). The area of the islets was determined from histological slides of insulin-stained pancreatic sections, using Leica Application Suite 3.1.0. (C) Representative stained slides of enlarged, insulin-positive islets from *RIP7-rtTA; tet-o-PyMT-IRES-Luc* bitransgenic mice that received doxycycline. Immunohistochemical staining for insulin, glucagon, somatostatin, and Ki67 in pancreatic sections from *RIP7-rtTA; tet-o-PyMT-IRES-Luc* bitransgenic mice that were not on doxycycline (un-induced, left panels) and from those that received doxycycline for 4 months (induced, right panels). Original magnification, 20×. The images are representative of more than 5 mice from each group.

We first determined the consequences of expressing the *PyMT-IRES-Luc* transgene exclusively in β cells of the pancreas. Milo-Landesman et al. (2001) previously described mice bearing a transgene, *RIP7-rtTA*, expressing the reverse tetracycline trans-activator protein (rtTA) in β cells under the control of the rat insulin promoter (RIP). These mice were crossed to our 6 founder lines, so that the *PyMT-IRES-Luc* transgene could be induced by doxycycline, an analog of tetracycline, specifically in β cells. Some of the resulting bitransgenic mice, were placed on a diet containing doxycycline at 4 weeks of age, monitored for the expression of the *PyMT-IRES-Luc* transgene by *in vivo* bioluminescent imaging weekly, and sacrificed at various ages for histological examination. We focused on *RIP7-rtTA; tet-o-PyMT-IRES-Luc* bitransgenic mice derived from 2 founder lines (#21 and 29), in which the transgene was tightly regulated, as indicated by bioluminescence (Figure 1A and data not shown). These bitransgenic mice exhibited luciferase activity (10^6^ to 10^7^ photons/sec) from the area over the pancreas after being placed on a diet containing doxycycline for 1 day. We observed some enlarged islets histologically after 2 weeks on doxycycline (data not shown). After 4 months on doxycycline, the bitransgenic mice still displayed luciferase activity (10^6^ to 10^7^ photons/sec; Figure 1A). None of *RIP-rtTA* and *tet-o-PyMT-IRES-Luc* mono-transgenic mice on doxycycline or the bitransgenic mice without doxycycline displayed detectable luciferase activity (Figure 1A). Immunohistochemical staining showed that the islets were enlarged 29.5-fold on average in *RIP7-rtTA; tet-o-PyMT-IRES-Luc* bitransgenic mice receiving doxycycline for 4 months, and most of the cells in the enlarged islets were positive for insulin (Figure 1B, C). Detailed histological examination revealed no abnormality in other tissues from *RIP7-rtTA; tet-o-PyMT-IRES-Luc* bitransgenic mice that received doxycycline for a year (data not shown).

To test whether the number of endocrine cells other than β cells also increased in the enlarged islets, we performed immunohistochemical staining with antisera against glucagon, somatostatin, and pancreatic polypeptide. α, δ, and pp cells persisted in the enlarged islets from the bitransgenic mice on doxycycline for 4 months, but their numbers were not greater than those in the normal islets from the bitransgenic mice without doxycycline (Figure 1C and data not shown). The results suggest that the enlarged islets result from PyMT-stimulated growth solely of insulin-expressing β cells.

Adult islet cells in un-induced control *RIP7-rtTA; tet-o-PyMT-IRES-Luc* bitransgenic mice had a low proliferation index of less than 0.5%, as determined by nuclear staining for the cell proliferation marker, Ki67 (Figure 1C). PyMT-induced β-cell hyperplasia was accompanied by a proliferation index of 3.7±1.5% (Figure 1C). Despite β-cell hyperplasia, the mice did not develop neoplasms or exhibit significant hypoglycemia (blood glucose levels = 176±41 mg/dL in 7 *RIP7-rtTA; tet-o-PyMT-IRES-Luc* bitransgenic mice on doxycycline for 1 year vs. 184±22 mg/dL in 6 age-matched non-transgenic mice). We conclude that PyMT is able to induce hyperplasia in β cells and that cells in the β cell lineage can respond to PyMT by undergoing proliferative changes with retained endocrine, rather than exocrine, features. The hyperplastic cells do not change normal homeostatic control of glucose levels and do not progress to frank malignancy of either endocrine or exocrine types.

In these initial experiments, the *PyMT-IRES-Luc* transgene was induced only after weaning. To test whether the effects of *PyMT* depended on the developmental stage of the β cell lineage at which it was expressed, we induced the oncogene at an early stage of pancreatic development. Animals mated to generate *RIP7-rtTA; tet-o-PyMT-IRES-Luc* bitransgenic mice received dietary doxycycline to allow induction of the transgene during pancreatic organogenesis throughout embryonic development in pregnant females; the resulting bitransgenic progeny were also maintained on doxycycline. The rat insulin promoter is normally activated around day E9.5 [11], and doxycycline is able to cross the placenta and to accumulate in the milk of lactating females [12]; we therefore presume that PyMT was produced in β cells during the latter half of embryogenesis and early postnatal development in bitransgenic progeny. However, when sacrificed at 6 months or 1 year of age, the pancreata of the bitransgenic progeny did not appear different histologically from those in bitransgenic animals that received doxycycline only after weaning; they exhibited enlarged hyperplastic islets composed of mostly insulin-positive β cells (6 mice at each time point, data not shown). Our results suggest that the *PyMT* oncogene cannot transdifferentiate β cells or any insulin-producing precursor to an exocrine phenotype or cause progressive neoplasia, regardless of the stage of development at which the oncogene is expressed.

In hopes of promoting tumorigenesis from β-cell hyperplasia, *RIP7-rtTA; tet-o-PyMT-IRES-Luc* bitransgenic mice were also crossed to *Ink4a/Arf*-null or *Arf*-null mice (n = 12, for each group). However, examination of histological pancreatic sections from these mice at 6 months of age that were kept on doxycycline revealed no β-cell tumor formation (data not shown).

### PyMT also induces β-cell hyperplasia when regulated by a *Pdx1-tTA* transgene

We were surprised to find that the highly potent PyMT oncoprotein failed to cause neoplastic changes in β cells. To attempt to confirm this finding and rule out the possibility that transdifferentiation to any non-β cell phenotype will repress the expression of PyMT from the rat insulin promoter, we took advantage of *Pdx1-tTA* knock-in mice, that express the tetracycline trans-activator protein (tTA, which acts positively on the tet operator in the absence of doxycycline) under the control of the endogenous pancreatic and duodenal homeobox 1 (*Pdx1*) promoter [13]. Pdx1-positive precursor cells of the early embryonic pancreas give rise to all epithelial lineages of the pancreas [14], but after birth *Pdx1* is expressed at high levels in β cells and at lower levels in some exocrine cells [15]. Moreover, Pdx1 is upregulated in PyMT-induced ductal and acinar tumors in *elastase-tva*; *Ink4a/Arf*-null transgenic mice [7], suggesting that the Pdx1 promoter remains active in the exocrine tumors.

To express *PyMT* postnatally in *Pdx1-tTA; tet-o-PyMT-IRES-Luc* mice, we placed mice pregnant with bi-transgenic progeny on doxycycline, then activated *PyMT* in the bitransgenic progeny at three weeks of age by removal of doxycycline. When these animals were sacrificed at 21 weeks of age for histological examination of the pancreas, we found β-cell hyperplasia indistinguishable from that observed in *RIP7-rtTA; tet-o-PyMT-IRES-Luc* mice on doxycycline and no other types of lesions were observed (data not shown). These findings confirm that the *PyMT* oncogene cannot transdifferentiate β cells to an exocrine phenotype, and that β cells have an intrinsically different response to PyMT than observed in mammary glands, endothelial cells, exocrine pancreas, and liver, in which *PyMT* acts as a strong oncogene [7], [16], [17].

### Induction of *PyMT* by Pdx1-tTA in pancreatic progenitor cells induces lethal acinar cell carcinomas, in addition to β-cell hyperplasia

**Figure 2:**
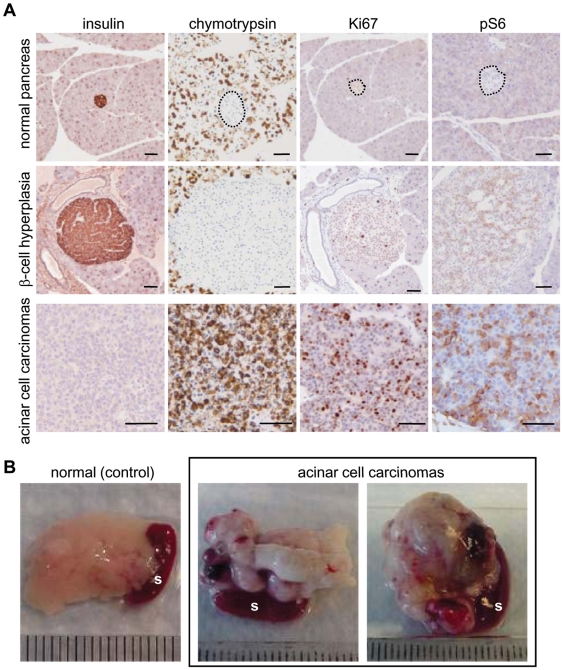
*Pdx1-tTA*; *tet-o-PyMT-IRES-Luc* bitransgenic mice develop β-cell hyperplasia or lethal acinar cell carcinomas when PyMT is expressed during and after pancreatic development. (A) Immunohistochemical staining was used to detect the expression of differentiation markers (insulin and chymotrypsin), Ki67, and phospho-S6 ribosomal protein (pS6) in normal pancreas, β-cell hyperplasia from 1-year-old *Pdx1-tTA*; *tet-o-PyMT-IRES-Luc* bitransgenic mice that were never on doxycycline (induced), and acinar cell carcinomas from 7 or 17-week-old *Pdx1-tTA*; *tet-o-PyMT-IRES-Luc* bitransgenic mice that were never on doxycycline (induced). Bar: 50 µm. (B) Representative normal pancreas and spleen from a non-transgenic animal (left) and acinar cell carcinomas and spleens from two 7.5-week-old *Pdx1-tTA*; *tet-o-PyMT-IRES-Luc* bitransgenic mice that were never on doxycycline (middle and right). s: spleen.

To ask whether PyMT could cause exocrine lesions as well as endocrine lesions when expressed starting from pancreatic progenitor cells, we took advantage of the fact that Pdx1-tTA is expressed in progenitor cells early in pancreatic development, as well as in both exocrine and endocrine compartments postnatally. Mice pregnant with *Pdx1-tTA; tet-o-PyMT-IRES-Luc* embryos and the resulting bitransgenic progeny (a cohort of 50 mice) were maintained on a doxycycline-free diet so that PyMT would be expressed throughout pancreatic development and postnatal life. These animals were tested for bioluminescence bi-weekly after 6 weeks of age, and formed two groups according to the strength of luciferase signals and types of pancreatic lesions (Figure 2A).

More than 90% of the *Pdx1-tTA; tet-o-PyMT-IRES-Luc* bitransgenic mice (45 of 50; ages between 4 months to 1 year) maintained on a doxycycline-free diet exhibited moderate luciferase activity (10^6^ to 10^7^ photons/sec) and had enlarged islets composed mainly of insulin-expressing β cells (Figure 2A middle row). The histological findings were similar to the β-cell hyperplasia observed when *PyMT* was induced postnatally in *Pdx1-tTA; tet-o-PyMT-IRES-Luc* mice and in *RIP7-rtTA; tet-o-PyMT-IRES-Luc* mice with doxycycline (data not shown). Again, when 12 of these mice were examined at 1 year of age, no frank islet neoplasms were found (data not shown).

In contrast, 4 of the 50 mice showed very high luciferase activity (>10^8^ photons/sec) and developed palpable and ultimately lethal pancreatic tumors at various times (7∼30 weeks of age; Figure 2B). These tumors had histologic features of acinar cell carcinomas and expressed chymotrypsin, an enzyme produced by acinar cells of the pancreas (Figure 2A third row), suggesting that PyMT had induced acinar cell carcinomas in these animals. These acinar cell neoplasms were histologically and immunohistochemically indistinguishable from their spontaneous human counterparts [18]. In contrast to the tumors induced by PyMT in the *elastase-tva*; *Ink4a/Arf*-null model of Lewis et al. [7], these acinar cell carcinomas in *Pdx1-tTA; tet-o-PyMT-IRES-Luc* mice completely lacked endocrine differentiation by immunohistochemistry. Furthermore, the tumors did not express insulin or a ductal marker, mucicarmine (Figure 2A and data not shown). These tumors were highly proliferative, with 46.7±12%, of the cells positive for Ki67 and tumor cells stained strongly for phospho-S6 ribosomal protein as revealed by immunohistochemistry (Figure 2A). Acinar cell neoplasms overwhelmed the entire pancreas, and normal pancreatic parenchyma including islets could not be found in these 4 animals. However, one other animal had relatively small acinar cell carcinomas co-existing with β-cell hyperplasia (data not shown).

### PyMT is not required to maintain the expanded β cell population

**Figure 3:**
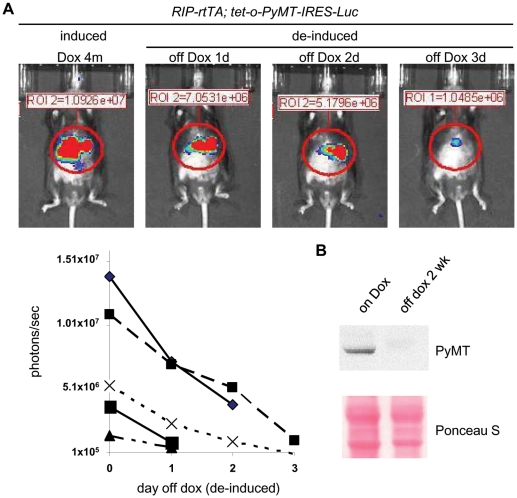
Reduction of luciferase activity and PyMT protein levels after withdrawal of doxycycline from the diet of *RIP7-rtTA; tet-o-PyMT-IRES-Luc* bitransgenic mice. (A) Studies with *RIP7-rtTA; tet-o-PyMT-IRES-Luc* bitransgenic mice that had been fed doxycycline for 4 months before doxycycline was removed for 1, 2, and 3 days. Upper panels show *in vivo* bioluminescent images acquired from a single *RIP7-rtTA; tet-o-PyMT-IRES-Luc* bitransgenic mouse. Lower panel shows results with 5 mice; each mouse is represented with a different symbol. (B) SDS-PAGE and Western blotting were used to determine the levels of PyMT proteins, detected with an antiserum against PyMT, in whole cell extracts of isolated islets of a *RIP7-rtTA; tet-o-PyMT-IRES-Luc* bitransgenic mouse that received doxycycline for 4 months (left lane; induced) and a bitransgenic mouse that had received doxycycline for 4 months and off doxycycline for 2 weeks (right lane; de-induced). A portion of nitrocellulose membrane stained with Ponceau S was shown as a loading control.

To determine whether PyMT has a role in maintaining, as well as in initiating, β-cell hyperplasia, we de-induced PyMT and luciferase by withdrawing doxycycline from the diet of *RIP7-rtTA; tet-o-PyMT-IRES-Luc* bitransgenic mice that had received doxycycline for 4 months. We initially monitored the activity of the luciferase reporter, using bioluminescence, and then performed Western blots on protein extracts from islets to document the levels of PyMT protein. We found that, within 3 days after removal of doxycycline, luciferase activity dramatically declined (Figure 3A), and no PyMT protein was detectable in the islets 2 weeks after removal of doxycycline (Figure 3B); these findings indicate that the expression of *PyMT* and *luciferase* was efficiently down-regulated.

**Figure 4:**
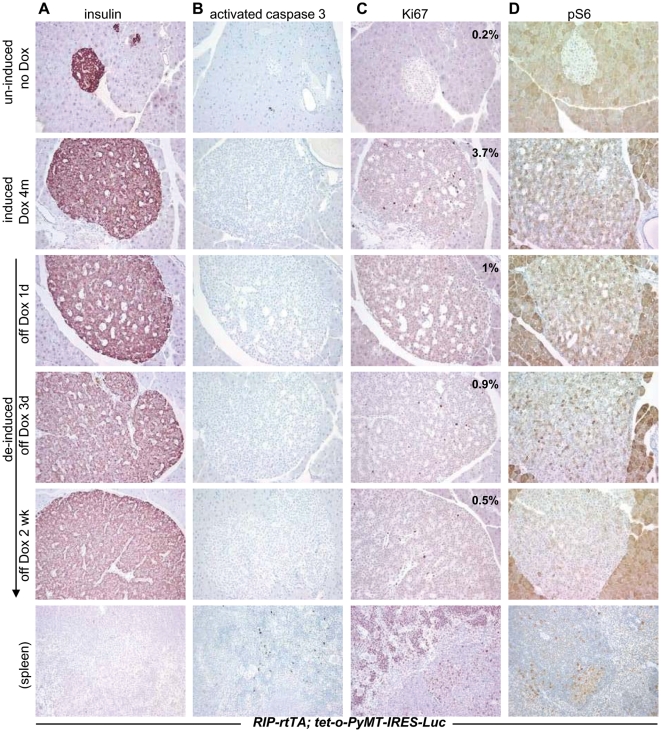
Effects of de-induction of *PyMT* on the enlarged islets of *RIP7-rtTA; tet-o-PyMT-IRES-Luc* bitransgenic mice. Sections of pancreata from *RIP7-rtTA; tet-o-PyMT-IRES-Luc* bitransgenic mice that were never on doxycycline (un-induced) or mice that were placed on doxycycline at 4 weeks of age for 4 months, with removal of the inducer for 1 day, 3 days, or 2 weeks, were stained with antibodies against insulin (A), activated caspase 3 (B), Ki67 (C), and pS6 (D). The proliferation indices of the islets indicated in (C) are the average of the percentage of Ki67^+^ cells from at least 5 islets under 40× magnification at each time point. Sections of spleens, which contain apoptotic cells, were used as a positive control for activated caspase 3 staining, at the bottom of the figure. Original magnification, 20×.

When the pancreata were examined histologically, however, enlarged islets composed of insulin-positive cells were still present 2 weeks after withdrawal of doxycycline (PyMT de-induced; Figure 4A). Furthermore, no apoptotic cells were detected in the enlarged islets by immunohistochemical staining for activated caspase 3 between 1 and 14 days following withdrawal of doxycycline (Figure 4B), indicated that the viability of the hyperplastic β cells was not dependent on continued production of the oncoprotein. However, cell proliferation and growth were diminished; cells in the enlarged islets stained less intensely with antisera against Ki67 and phospho-S6 ribosomal protein (Figure 4 C, D). The islets remained enlarged for at least three months after withdrawal of doxycycline (data not shown), further suggesting that hyperplastic β cells do not require PyMT to remain viable, even though PyMT is required for continued proliferation. In addition, the blood glucose levels of these mice did not change significantly after withdrawal of doxycycline (data not shown), indicating that PyMT did not modulate the expression of rtTA or insulin.

We confirmed these unexpected findings of oncogene-independent survival of the hyperplastic cells using *Pdx1-tTA; tet-o-PyMT-IRES-Luc* bitransgenic mice. Twelve such mice with moderate luciferase activity (10^6^ to 10^7^ photons/sec) on a doxycycline-free diet were given doxycycline to turn off the expression of transgene (de-induced), and then imaged and sacrificed 1 day to 2 months later. After a 1-day of de-induction, luciferase activity returned to background levels as measured by bioluminescence, but enlarged β-cell islets remained up to at least 2 months after de-induction (data not shown). Thus, regardless of whether *Pdx1-tTA* or *RIP-rtTA* is used to regulate expression of the inducible *PyMT*, continued expression of *PyMT* is not required for maintenance of the expanded β cell population, only for persistent growth.

### PyMT is required for maintenance of acinar carcinomas in *Pdx1-tTA*; *tet-o-PyMT-IRES-Luc* bitransgenic mice

**Figure 5:**
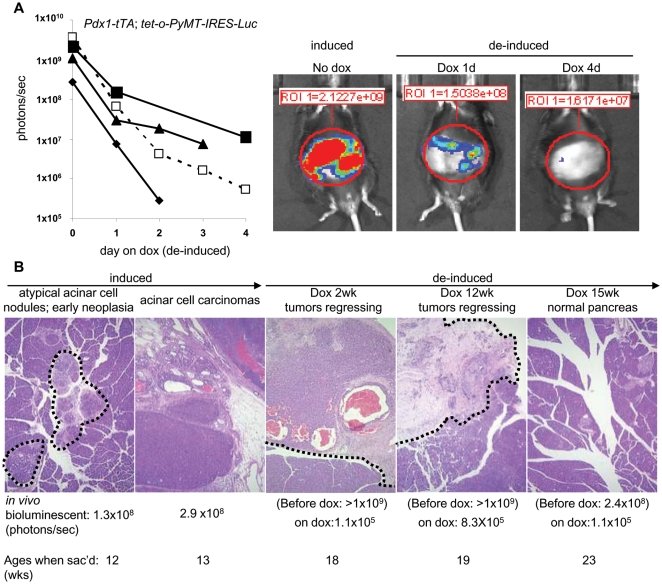
Effects of de-induction of *PyMT* and *luciferase* in the acinar cell carcinomas of *Pdx1-tTA*; *tet-o-PyMT-IRES-Luc* bitransgenic mice. (A) Bioluminescence intensities from 4 *Pdx1-tTA*; *tet-o-PyMT-IRES-Luc* bitransgenic mice before and after being placed on doxycycline for 1 to 4 days; each symbol denotes a different mouse. Bioluminescent images of one mouse (represented by ▪) are shown on the right during induction and 1 and 4 days after addition of doxycycline. (B) De-induction of *PyMT* in *Pdx1-tTA*; *tet-o-PyMT-IRES-Luc* bitransgenic mice with acinar cell carcinomas causes tumor regression. Hematoxylin and eosin stain of pancreatic sections from *Pdx1-tTA*; *tet-o-PyMT-IRES-Luc* bitransgenic mice with strong luciferase activity (>10^8^ photons/sec) before and after being placed on doxycycline for 2, 12, and 15 weeks. Original magnification, 5×. The ages of the mice when sacrificed were indicated.

PyMT and other oncoproteins are known to be required to maintain the viability of cancer cells they induce [9], [10]. In view of our findings with PyMT-induced β-cell hyperplasia, we determined whether the survival or proliferation of PyMT-induced acinar cell carcinoma cells depended on continued expression of *PyMT*. To do this, we identified 8 *Pdx1-tTA*; *tet-o-PyMT-IRES-Luc* bitransgenic mice with strong luciferase activity (>10^8^ photons/sec) and palpable pancreatic acinar cell carcinomas, and placed them on a doxycycline-containing diet to turn off the expression of the *PyMT-IRES-Luc* transgene. Mice were imaged and sacrificed 1 day to 15 weeks later to assess the response of tumor cells to de-induction of *PyMT*. One to 4 days after de-induction, we observed a large reduction of luciferase activity by bioluminescence (Figure 5A). Two weeks after de-induction, the acinar neoplasms were no longer palpable. The tumors were still grossly visible when mice were sacrificed at this time (Figure 5B, third panel), but they gradually regressed and the pancreata returned to a normal histological appearance over the course of 15 weeks (Figure 5B, fifth panel). Mostly normal-sized islets were observed in the regenerated pancreata after the acinar cell carcinomas regressed. Apoptotic cells, as identified by the TUNEL assay, and loss of Ki67^+^ cells were observed in the regressing acinar cell carcinomas (data not shown). Our observations, in concert with many published studies [9], [10], strongly suggest that PyMT is required to maintain cell viability in PyMT-induced pancreatic acinar cell carcinomas.

## Discussion

We have generated tetracycline-inducible *PyMT* transgenic mice to investigate the consequence of activating *PyMT* oncogene in different cell types in the pancreas and at different developmental stages. To facilitate transgene detection *in vivo*, we placed a *luciferase* reporter gene behind IRES downstream of *PyMT*. Induction of *PyMT* in the pancreatic β cell lineage in *RIP7-rtTA; tet-o-PyMT-IRES-Luc* bitransgenic mice resulted in β-cell hyperplasia regardless of their developmental stages. β-cell hyperplasia was also induced in *Pdx1-tTA*; *tet-o-PyMT-IRES-Luc* bitransgenic mice, and the insulin-expressing β-cell hyperplasia does not further progress to form frank neoplasms even after 1 year of oncogene activation and in *Ink4a/Arf*-null and *Arf*-null backgrounds. Upon de-induction of *PyMT*, the proliferation was greatly reduced in these hyperplastic islets. Nevertheless, no apoptotic β cells were detected at any time during induction or de-induction of *PyMT*, and the islets remained enlarged once they were expanded, even when *PyMT* was de-induced.

A few (less than 10%) of *Pdx1-tTA*; *tet-o-PyMT-IRES-Luc* bitransgenic mice developed lethal acinar cell carcinomas in adulthood when the *PyMT* transgene was induced during embryonic pancreatic development. These acinar cell carcinomas resemble their spontaneous human counterparts. Because no exocrine tumors were observed when the *PyMT* transgene was induced postnatally, we concluded that early precursor cells in the embryonic pancreas are likely to be the source of the acinar cell carcinomas. One *Pdx1-tTA*; *tet-o-PyMT-IRES-Luc* mouse with acinar cell carcinomas also showed β-cell hyperplasia, but in most cases acinar cell carcinomas apparently arose sooner than β-cell hyperplasia and overwhelmed the whole pancreas. Unlike PyMT-induced β-cell hyperplasia, continued expression of *PyMT* is required to maintain the viability of the acinar cell carcinomas. After *PyMT* de-induction, acinar tumor cells underwent apoptosis and stopped proliferation, and the pancreas gradually returned to its normal appearance over 15 weeks. Interestingly, most of the islets were not enlarged in the pancreata after regression of the huge acinar cell carcinomas, indicating that pancreata were newly regenerated.

In the *Pdx1-tTA*; *tet-o-PyMT-IRES-Luc* bitransgenic mice that have never received doxycycline, all epithelial lineages of the pancreas express *PyMT* during development. However, neither non-β-cell endocrine neoplasms nor PanINs have been observed, even though Pdx1^+^ precursors give rise to these cells, and other oncogene models targeting Pdx1^+^ precursors produce PanINs and infiltrating ductal adenocarcinomas [19]. Our results suggest that transient expression of *PyMT* is not adequate for tumor initiation in α, δ, ε, pp, and ductal cells.

Production of PyMT in transgenic mice or through retroviral delivery has been shown to lead to tumor formation in mammary glands, endothelial cells, liver, and pancreatic exocrine cells [7], [16], [17]. The potency of PyMT is attributed, at least in part, to its ability to activate MAPK and PI3K/Akt signaling pathways [8]. It has been shown that PI3K/Akt signaling pathway affects β cell size and function. Constitutive activation of *Akt1* in β cells increases cell size and decreases blood glucose levels [20], [21]. Selective deletion of the phosphatase and tensin homologue, PTEN, an antagonist of PI3K signaling, in β cells results in a 4.5-fold increase in total islet area by increasing both cell size and number [22], [23]. Compared to these models, PyMT induces more extensive β-cell hyperplasia with a ∼30-fold increase in islet area without significant change in cell size and blood glucose level, and mice do not develop malignant β-cell tumors. It is possible that some of the signaling molecules modulated by PyMT in the course of tumorigenesis are not expressed or active in β cells.

Although induction of *PyMT* does not lead to tumorigenesis in β cells, even in *Ink4a/Arf*-null and *Arf*-null backgrounds, β cells are subject to transformation by other oncogenes, such as SV40 T antigen (Tag) and Myc; in addition, the viability of either the hyperplastic or tumor cells is dependent on the continued expression of the initiating oncogene [24]–[26].

Tag provides the driving force for tumor initiation by blocking the activities of Rb and p53 tumor suppressors [27]. The process of Tag-induced islet hyperplasia is accompanied by apoptosis, but β cells generated by proliferation outnumber the apoptotic cells [28]. Importantly, the islet hyperplasia is reversible upon de-induction of Tag in *RIP7-rtTA; tet-o-Tag* bitransgenic mice, and the islets are gradually restored to their normal sizes through apoptosis within 4–8 weeks after doxycycline removal [24], an outcome different from our observation in PyMT-induced β-cell hyperplasia.

Activation of Myc in β cells initially causes rapid and synchronous entry into the cell cycle, but proliferation is overwhelmed by concomitant induction of apoptosis in transgenic mice [26]. When Myc is activated in islets that overexpress an anti-apoptotic molecule, Bcl-xL, β-cell hyperplasia is induced within 7 days and multiple, large invasive β-cell tumors develop after 6 weeks of Myc activation. Both the hyperplasia and the tumors rapidly regress by apoptosis when Myc is inactivated, indicating that the survival of these Myc-expanded cell populations is dependent on continued Myc activity.

Our results with induction and de-induction of *PyMT* in β cells differ from results in experiments with several other inducible transgenic models, affecting various tissues, in which continued activity of the initiating oncogenes is necessary to maintain cell viability. Even the survival of metastases, the most advanced stages of tumorigenesis, has been shown to depend on expression of the initiating oncogene [29].

Several possibilities might explain why the expanded β cells induced by PyMT do not regress upon de-induction of *PyMT*. First, expression of *PyMT* may trigger or be accompanied by additional mutations, epigenetic changes, or alterations in the microvasculature that are able to prevent regression. Second, β cells in adult mice have been shown to arise from pre-existing β cells, not from stem cells [30]. It is likely that PyMT induces hyperplastic islets through a mechanism similar to β cell duplication and that these duplicated, differentiated cells do not depend on the continued expression of *PyMT* for their viability. Third, PyMT-induced β-cell hyperplasia has a low proliferation index (as judged by Ki67 staining: 3.7±1.5%), compared to acinar cell carcinomas (46.7±12%), Tag-induced β-cell hyperplasia (21.1±3%; data not shown), and Myc-induced β-cell hyperplasia (near 100%) [26]. It is possible that the oncogenic signaling pathways are dispensable for the survival of slowly proliferating cells, whereas they are required for the survival of the highly proliferative cells.

In summary, we have shown that PyMT can induce oncogene-dependent exocrine tumors from immature pancreatic cells, but expression of *PyMT* in the β cell lineage leads to an irreversible increase in β-cell numbers. Importantly, PyMT-induced β-cell hyperplasia does not progress to malignant tumor formation. Blood glucose levels in mice with PyMT-induced β-cell hyperplasia are within normal ranges, indicating the preservation of intact glucose-sensing capacity in the expanded β-cell population. Conceivably, understanding the mechanism by which β cells proliferate in response to PyMT and how expanded islets are maintained after de-induction of the oncogene might help to develop strategies to enhance human islet cells expansion *in vitro* for transplantation in diabetic patients.

## Materials and Methods

### Mouse strains, animal husbandry, and genotyping

*PyMT* DNA (∼1.27 kb) was amplified from pBluescript II-*PyMT* by PCR using primers containing *EcoR*I and *Cla*I sites, and was then released by *EcoR*I/*Cla*I digestion and subcloned into a *EcoR*I/*Cla*I digested TMILA plasmid which contains the tetracycline operator, an internal ribosome entry site (IRES), firefly luciferase coding region (Luc), and the SV40 polyadenylation signal [29], [31]. The resulting 5.8 kb *tet-o-PyMT-IRES-Luc* transgene construct was released from the TMILA vector backbone by digestion with *Not*I, purified, and resuspended in TE for pronuclear injection. The *tet-o-PyMT-IRES-Luc* transgenic founders were generated on a pure C57BL/6 genetic background. *RIP7-rtTA* mice [32] were rederivatived onto a pure C57BL/6 genetic background. *Pdx1-tTA* knock-in mice were on an ICR genetic background [13]. Doxycycline was administered by feeding mice with doxycycline-impregnated food pellets (625 ppm, Harlan-Teklad). Mice were housed in accordance with Memorial Sloan-Kettering Cancer Center (MSKCC) institutional guidelines and all experiments involving mice were approved by the Institutional Animal Care and Use Committee at MSKCC. Genotypes were determined by PCR using mouse tail DNA prepared using Qiaprep Tail DNeasy isolation kit (QIAGEN) according to the manufacturer's instruction. PCR primers were: for *PyMT*: 5′-CTGCTACTGCACCCAGACAA-3′ and 5′-TCCGCCGTTTTGGATTATAC-3′ (a 468-bp product); for *rtTA*: 5′-GTGAAGTGGGTCCGCGTACAG-3′ and 5′-GTACTCGTCAATTCCAAGGGCTCG-3′ (a ∼450-bp product); and for *tTA*: 5′- TAGAAGGGGAAAGCTGGCAAGATT-3′ and 5′-CCGCGGGGAGAAAGGACA-3′ (a 540-bp product).

### *in vivo* bioluminescent imaging

Mice were anesthetized with 3% isoflurane and with shaved abdomen received a retro-orbital injection with 50 µl of 30 mg/ml D-luciferin (Caliper Life Sciences, XR-1001). Images were acquired for 2 min using Xenogen IVIS 100 system with Living Image 2.50 software. A photographic image of the mouse was taken, onto which the pseudocolor image representing the spatial distribution of photon counts was projected. We defined a circular region around the pancreas and used it as a standard in all experiments. From the region, the photo counts were compared among mice.

### Histology and immunohistochemical analysis

Tissues were removed and fixed in 10% buffered formalin overnight at room temperature. Fixed tissues were processed and cut into 5 µm sections at Histoserv Inc. (Germantown, MD). Formalin-fixed/paraffin-embedded sections were deparaffinized and rehydrated by passage through a graded xylene/ethanol series before staining. Immunochemistry with VECTASTAIN Elite ABC Kit (Vector Laboratories) was performed according to the manufacturer's instructions. Primary antibodies used were anti-insulin (Immunostar, 1∶600), anti-synaptophysin (DAKO, 1∶100), anti-glucagon (Novocastra, 1∶1,000), anti-somatostatin (DAKO, 1∶10,000), anti-activated caspase 3 (Cell Signaling, 1∶100), anti-Ki67 (Novocastra, 1∶1,000), anti-phospho-Erk (Cell Signaling, 1∶100), anti-Erk (Cell Signaling, 1∶100), anti-phospho-Akt (Cell Signaling, 1∶100), and anti-phospho-S6 ribosomal protein (Cell Signaling, 1∶200).

### Western blot analysis

Pancreata were perfused and digested into small pieces with collagenase (Sigma) at 37°C for 15 min, and were centrifuged in a Ficoll gradient (11%, 20.5%, 23%, and 25% (w/v)) as described [33]. Islets in the top three layers of the gradient were collected. To prepare protein extracts, islets were homogenized with pellet pestles (Kontes) in Eppendorf tubes and lysed in buffer containing 100 mM NaCl, 100 mM Tris (pH 8.2), 0.5% NP-40, and inhibitor cocktail for proteases and phosphatases at 4°C. The cellular debris was pelleted at the top speed in a microcentrifuge for 10 min at 4°C, and the supernatant was transferred to new tubes. 20 µg total proteins were separated by SDS-PAGE and transferred onto nitrocellulose membranes. The membranes were stained with Ponceau S and immunoblotted with the following antibodies: anti-PyMT (1∶5,000), anti-phospho-Erk (Cell Signaling, 1∶1,000), anti-Erk (Cell Signaling, 1∶1,000), anti-phospho-Akt (Cell Signaling, 1∶250), anti-Akt (Cell Signaling, 1∶1,000), and appropriate secondary antibodies. The signals were detected with ECL chemiluminescent substrate (GE Healthcare).
